# Becoming, sensibility, and imagination in social research. A collaborative exploration with defenders of water in Chile

**DOI:** 10.3389/fsoc.2026.1813831

**Published:** 2026-07-10

**Authors:** María Consuelo Sánchez, Hugo Marcelo Zunino, Martin Araneda, Carla Cepeda

**Affiliations:** Universidad de La Frontera, Temuco, Chile

**Keywords:** becoming, Chile, collaborative exploration, imagination, sensitivity

## Abstract

In the framework of recent critiques of modern epistemologies that dichotomize sentient human beings from the world, this article explores modes of knowledge grounded in the interconnected becoming of human beings, other living beings, society, and nature. Drawing on the insights of Gilles Deleuze, Johann Wolfgang von Goethe, and Merleau-Ponty, among others, we develop a mode of social inquiry that approaches knowledge as a creative and collective endeavor activated as human beings enter into a sensible and imaginative relationship with the world. These ideas are put to work in a collaborative exploration with defenders of the waters of Lake Villarrica in Chile, with the aim of projecting this defense into the future. We employ body movement and artistic expression as tools to bridge the human-nature divide and herein create a collective space open to creation and exploration. The experience of becoming was partially achieved at specific junctures during the process, making it possible to recognize the intrinsic power of water and, from this collective realization, allow different perspectives to converge with renewed impulses. We highlight that the collective impulses for action are partial and unfinished achievements, always renewing themselves through the living interaction among diverse human beings.

## 1 Introduction: from abstractions to intertwining with the world

The purpose of this article is to develop and put to work an approach to social inquiry based on the principle that human beings are intertwined with the world. We understand this relationship in two related ways. On the one hand, nothing is given or static in the world; rather, everything is interconnected and in perpetual transformation, endlessly repeating and differentiating itself on a virtual plane. In this incessant movement, the distinction between subject and object ceases to have any meaning (Deleuze, 1994). On the other hand, when the human body interacts with the environment, meaning emerges, establishing an indissoluble relationship between experience and the world. Thus, perceptions and sensations are not simply mechanical responses to external stimuli but something of the world expresses itself in them (Merleau-Ponty, 1993). By bringing these ideas to empirical research, we respond to the abstract and mechanistic way of understanding the world, contributing to efforts to destabilize hegemonic ways of knowing (Escobar, 2015; Guattari, 2000; Haraway, 2019; Latour, 2007; Rivera-Cusicanqui, 2018; Shiva, 1995).

The basis for our contemporary way of conceiving the construction of knowledge can be traced to the classical thinkers of the intellectual Renaissance of the 16th and 17th centuries. Descartes (2005) separates the soul from the body and places the rational subject as the center of the world. In turn, Kant (2004) moves toward methodological dualism that limits our knowledge to our subjective representations, closing access to the “things in themselves.” The articulation of this idea created a chasm between humans and the world, reducing nature to a mere resource that could be exploited. It has provided society with powerful tools to comprehend the causal relationships governing the sensory world, thereby contributing to the significant material and social advancements that have occurred over the past few centuries. We invite the reader to observe and experience the methodological isolation imposed by this mode of thought. It dwells within us and stealthily colonizes us: we perceive the external world, weigh, measure, and count it, and then abstract laws that allow us to manipulate it. To predict is to know, says Comte (1965), transforming knowledge into a soulless device aimed at analyzing and categorizing the world around us.

The configuration of contemporary culture and society largely depends on this abstract intellect. In particular, it significantly influences contemporary technocracies, wherein societal demands are addressed through the expertise of specialists or by using machines and algorithms. As a result, issues are frequently reduced to administrative and technical concerns, thereby depoliticizing social movements and rendering them vulnerable to state control and manipulation (Accetti, 2021; Friedman, 2019). In what ways can we contribute to defining more participatory and self-sustaining grounds to support individuals in confronting the effects of developmental trends? Escobar (2015) suggests that contemporary problems cannot be solved by a social theory that rests on a dualistic epistemology that presupposes autonomous subjects and a reality that exists objectively and independently of us.

The limitations of classical epistemology have been recognized from various interconnected perspectives, leading to the proposal of alternative paths to knowledge. Decolonial literature emphasizes subaltern forms of knowledge that challenge Eurocentric power and knowledge structures (Grosfoguel, 2007; Quijano, 1992). The feminist perspective has introduced ways of knowing rooted in lived experiences, recognizing the diverse power dynamics involved in knowledge production (Anderson, 2024; Harding, 2011). In Latin America, community feminism highlights the inseparability of bodily and territorial experiences, fostering embodied and situated perspectives to co-construct knowledge and address conditions of dispossession and inequality (Cruz-Hernández, 2016; González et al., 2020; Haesbaert, 2020; Smith et al., 2016; Zaragocin and Caretta, 2020). Indigenous knowledge, in turn, emphasizes the interconnectedness of humans and nature and develops ways of understanding reality based on the hidden language of nature (Aikenhead and Ogawa, 2007; Quintriqueo and Arias-Ortega, 2019). Similarly, for Abram (1996), the sensual world envelops us in a reciprocal relationship with nature, establishing a connection between cognition and the living environment.

In this research, we contribute to the ongoing efforts to develop new ways of understanding our relationship with the social and natural environments, offering a research approach that emphasizes experiencing a way of thinking aligned with the incessant movement and transformation that characterizes the world. To formulate our perspective, we rethink and articulate ideas from Deleuze (1994, 2007), Deleuze and Guattari (1993, 2004), Goethe (1988, 2007), and Merleau-Ponty (1993). As discussed below, these thinkers, each from their own position and context, help us conceive and work within a research space situated between the realm of the sensible and the forces that sustain the becoming of the world. In this space, thinking loses its abstraction and connects to human experiences, gradually building a collective space of inquiry through which we can explore new ways of relating to the world. Various embodied and artistic tools can be utilized to enrich our experiences of interconnectedness and support the research process (see Section 3). Thus, our research establishes a connection between epistemology, methodology, and experience, complementing the efforts of decolonial, feminist, and indigenous perspectives to reconsider how knowledge is constructed.

The ideas we develop here are put to work in a collaborative exploration with people who mobilize to defend the waters from the aggression of capital in southern Chile, at a time marked by frustration and defeat following the social uprising of 2019–2020 (Frei, 2024). Recognizing the limitations of classical thought in addressing environmental problems in depth, the case study provides an epistemic moment to experience the ontological unity between human beings and nature, penetrating into the intimate relationship humans maintain with water (Neimanis, 2017a,b; Zaragocin and Caretta, 2020). From this foundation, we aim to visualize alternative ways of thinking about the problem, fostering a space for deep collective reflection that enables the discovery of new impulses for the defense of the waters. We use drawing, poetry, and the synchronization of bodies in motion as tools to cultivate a sense of interconnection with others and with nature. These practices provide experiences that stimulate imagination and encourage collective reflection. We embrace a flexible and open approach to social research in which researchers are actively involved in the investigative process.

In the following section, we articulate a mode of social inquiry based on the principle that human beings are fundamentally immersed in a world that ceaselessly transforms itself into something new. We propose that experiencing this becoming enables entry into a sphere filled with vital forces and creative potential. We then introduce the case study and outline the methods employed in our collaborative exploration. Next, we describe the results achieved in three workshops we characterize as “Difference in Motion,” “Intertwining with Water,” and “Hope and Future.” Subsequently, we examine the extent to which the methods used facilitate the experience of becoming and fostered new collective impulses for action. In the concluding section, we synthesize our achievements and reflect on the nature of this type of endeavor, highlighting the fragility of the social arrangement reached.

## 2 Knowledge as a creative event that emerges from the experience of becoming

Nietzsche (1962) asserts that pre-Socratic Greek philosophers perceived the universal logos acting within them. For Thales of Miletus, water is the origin of all things, embodying the unity of the cosmos and the essence of life. For Anaximander, air is the unifying principle, whereas, for Heraclitus, it is fire. In contrast, Parmenides recognizes the realm of cosmic ideas as the only reality and depicted things perceived by our senses as mere masks that obscure the true essence of the world. Parmenides perspective was further developed by Platón (1992, p. 193–197) in his Allegory of the Cave, which portrays human beings as prisoners confined to a cave, able only to perceive the projected silhouettes of the ideal world, the only reality they recognize.

Gilles Deleuze radically reconsiders the ancient approach to the wholeness of the world and places it in the continuous process of transformation that characterizes the sensory domain, moving from a transcendent to an immanent perspective. Indeed, it is no longer a universal logos situated beyond that guides the workings of human beings and nature. Instead, it is thought, as a living force, that expresses itself in the ever-transforming sensible world and enters into relation with the human being. He expresses this idea in the following:

*Something in the world forces us to think. This something is an object not of recognition but of a fundamental encounter. What is encountered may be Socrates, a temple or a demon. It may be grasped in a range of affective tones: wonder, love, hatred, suffering. In whichever tone, its primary characteristic is that it can only be sensed. In this sense it is opposed to recognition”* (Deleuze, 1994, p. 139).

Thus, in encountering the world, there is no subject attaching a certain concept to the object and recognizing it, but something is affecting us, and this something can only be “sensed.” Thinking is not an activity of the subject; rather, it is something that happens to the subject when the world resonates in us.

In this fundamental encounter with the world, then, we can distinguish two planes: the virtual and the actual. The actual plan consists of all objectively realized phenomena that are related to a virtual plane, from which all tangible forms appear through the process of actualization. The virtual is a plane of incessant movement, a plane of latent power, which is pre-philosophical, prior to any sensible manifestation. The virtual permeates the whole of existence, setting in motion a continuous process of difference and repetition that pervades all sensible phenomena, establishing an infinite series of events that articulate the radical interconnectedness of the world (Deleuze, 2007; Deleuze and Guattari, 1993). We can picture this virtual plane if we imagine a surfer riding the waves: the surfer does not struggle to control the movements or direction but rather lets himself be carried away by pure movement; the subject *becomes* the wave, as the subject and the wave are part of the same movement (Deleuze, 1995). The entire world is thinking in me and guiding my movements. In this image, everything static vanishes, and a plane of infinite possibilities emerges. Upon entering this virtual plane, people find themselves moving within a creative space, in which a collective wave may form that, through an upward spiral, leads to the creation of something new. Of course, the wave will break, and another will emerge; all knowledge becomes unstable and provisional.

A Deleuzian perspective challenges modern cognitive boundaries, dissolving all that is permanent in an incessant flow of transformation in which everything stable is always becoming something different (Deleuze, 1994). Thus, it rejects static categories and fixed identities and privileges multiplicity. A Deleuzian approach to knowing involves entering a creative flux of potentialities, characterized by a rhizomatic thinking that remains open, allowing diverse concepts and ideas to continuously metamorphose and actualize in novel ways (Deleuze and Guattari, 2004). This approach to knowledge raises the challenge of recovering the creative capacity that remains concealed from individuals, subjugating them to external authority (Campillos, 2024).

Like Deleuze, in his *Theory of Nature*, Goethe, when confronting the world, places the attention on differences rather than similarities. He contemplated natural phenomena without preconceived theories or hypotheses, observing and considering with an attentive mind the conditions under which differentiation occurs. The rigorous and systematic practice of this way of observing leads to an intuitive and artistic understanding, complementing what the senses perceive. He describes this approach as follows:

“My thought is not separate from the objects, but rather the elements of the object, its sensory images, converge in it; my observing is already thinking, and my thinking is observing” (Goethe, 2007, p. 211, translated by authors).

In the same work, Goethe invites us to perceive in nature and within ourselves an eternal becoming, a perpetual movement without a moment of stillness (p. 238). At this juncture, between the becoming of nature and that of human beings, knowledge presents itself to the observer as an image that refers us to an ideal principle active in nature and shaping its forms. This is the *Ur-phenomenon* (*Urphänomen*) or primal phenomenon (Böhme, 1986).

Thus, Goethean phenomenology looks for the principles and forces that are active in the phenomenon we perceive with our sense. The true being of a plant, from its germination to its death, is not found in the fixed facts observable with our senses, but is revealed as a process, a movement, a ceaseless transformation from one form to another (Simms, 2005, p. 167). Similarly, every triangle perceived through the senses as an object refers back to the ideal form “triangle,” which contains all possible tangible triangles and is always in motion (Böhme, 1986). This domain of the ideal establishes a relationship with the observable world similar to Deleuze's conception of the virtual and the actual. Goethe does not seek a transcendental force or being situated in the beyond as the foundation of changing forms; instead, he remains faithful to what is perceived by the bodily senses and seeks within the phenomenon itself the expression of the ideal principles at work, thereby establishing an immanent perspective of inquiry in which the world is revealed in and through the human being (Muenzer, 2006).

The immanent path outlined by Goethe leads to a form of knowledge that can be described as a dialogue between the observer and the world around. In this meeting, a mutual interaction with phenomena is established, revealing how the principles and forces intrinsic no nature also manifest within the human being. This requires the observer to set aside abstract speculation and face the world from an inner emptiness, which in turn is a state of full attention to the phenomenon being observed (Holdrege, 2005). To experience what happens within us, it is necessary to develop the faculty of imagination. Imagination (Einbildungskraft) can be understood as a mediating force between the capacity to think (Denkkraft) and the internal elaboration of perceptions and affects, thereby producing images (Darstellungen) that intertwine the inner world of human beings with the world perceived through the senses (Pulvirenti and Gambino, 2022). In this way, at the moment of encountering the world, our inner being reacts, giving rise to imaginations that allow us to complete what we perceive with the senses, giving the world coherence and meaning (Hüppauf and Wulf, 2009, p. 13–14). Goethe's ideas provide an empirical way of exploring formative or ideal forms active in nature and society, activating a way of thinking that is also a way of observing (Kaplan, 2015).

The work of French phenomenologist Maurice Merleau-Ponty can help us observe more clearly the articulation between what is perceived and felt within the inner being and the events that occur in the process of becoming. In contrast to the modern idea that our perceptions are produced within us from stimuli we receive from the external world, Merleau-Ponty (1993) argues that the body is not a passive entity that merely receives information from the outside but rather actively anticipates perception, synchronizing with the meaning of the world, thereby establishing a reciprocal relationship between the embodied subject and the world. While the body naturally understands its connection to the world, the abstract intellect separates us from the world by directing our attention to the object of perception rather than to what the body experiences during the act of perceiving. These elements open up a living and experiential way of knowing, focusing on what happens in the body when it encounters the world. To illustrate this relationship, Merleau-Ponty provides an example of dance. Learning this skill does not require abstract intellectual work, rather, it emerges from a reciprocal relationship we establish with the melodies we perceive, at the same time as our body is engaged and active in generating movement. Just as Deleuze imagines a surfer riding the waves on the plane of immanence, Merleau-Ponty leads us to observe, in dance, an event in which the external and internal worlds are interwoven in an incessant movement that is full of meaning.

Scholarly work has considered the relationships among the ontologies of Gilles Deleuze, Johann Wolfgang von Goethe, and Merleau-Ponty, highlighting how they resonate and enrich one another (Amrine, 2015; Montenegro, 2010; Shores, 2012). Our interest here is to move from epistemological debates to the concrete application of these ideas in empirical settings. These three perspectives share a radical interdependence between humans and the world, offering valuable concepts and tools for investigating how the surrounding atmosphere embraces and absorbs us in its ceaseless, creative movement. This vital interconnection with the world has been characterized as a virtual field of intensities in a state of potential (Deleuze, 1994; Deleuze and Guattari, 2004), as an ideal principle that permeates sensible forms and can be grasped as an imagination (Goethe, 2007), and as the irrevocable presence of meaning embedded in any perception (Merleau-Ponty, 1993). Despite the diverse ways and angles of presenting it, we are driven to recognize the presence of a force manifesting in the continuous transformation of sensory forms that never reach a state of rest. We will refer to this state of transformation as the becoming of the world, employing this expression in broad terms and granting it sufficient flexibility to apply it to concrete empirical settings. From this perspective, the act of knowing appears very different from the classical mode of thought we have inherited. Knowing manifests as an event that unfolds as individuals intertwine with the becoming of the world, as bodies are affected by myriad forces imbuing them from all sides. Thus, knowing emerges from the vital and creative relationship that our bodies establish with the world, not from an abstract reflection of a world that exists independently of us.

To progress in experiencing becoming, it is essential to disrupt the abstract and mechanistic thought patterns that dominate modern social life, allowing the body to be affected by the dynamic flow of the world around us. Various approaches exist for experiencing this domain. From a Deleuzian perspective, the importance of desire and artistic creativity is emphasized as a means of accessing a virtual plane of infinite possibilities (Larrauri, 2007; Pérez de Lama, 2009; Semetsky, 2004; Torres, 2018). Contemporary Goethean scholars have concentrated on introspective imaginative practices to reach the ideal forces active in phenomena (Brook, 1998; Gosetti-Ferencei, 2025; Pulvirenti and Gambino, 2013; Seamon, 2012; Seamon and Zajonc, 1998). From Merleau-Ponty's perspective, the focus shifts to the capacity of the human body to capture the meaning inherent in the world (Abram, 1996; Purser, 2018; Thorndahl and Ravn, 2016). Within this comprehensive range of approaches that can be put to work to experience becoming, we aim here to integrate the Deleuzian mode of investigation through the expression of creativity and desires with a more introspective Goethean perspective, while also giving due consideration to the role of the body as a crucial mediator between humans and the world.

In the collaborative exploration we will undertake with individuals defending the waters in southern Chile, we aim to experience together with them the becoming of the world and, from that position, create a collective space to project this defense into the future. Water provides a unique opportunity to experience becoming, as it is more than a discrete element; it is a vital force that closely binds us to the world. Indeed, water is the main component of the human body, sustaining life and intertwining us through its continuous flow with other watery bodies and more-than-human worlds (Neimanis, 2017a). The material and ontological relationship that human beings establish with water offers us an imaginative and epistemic space where we can think with water and not about it, thus transforming water into an interlocutor on how we can make sense of the world by experiencing our interconnectedness with her (Neimanis, 2017b). In our exploration, we used drawing, poetry, and the synchronization of bodies in motion as tools to experience becoming and stimulate the faculty of imagination, situating ourselves between the actual and virtual planes to grasp how the forces of the world resonate within us. Experiencing this investigative space allows us to explore a mode of thinking that is no longer abstract but moves through a plane of vital activity imbued with creative potential.

## 3 Case-study and methodological approach

Lake Villarrica in Chile is located in the foothills of the Andes, approximately 800 km south of Santiago, the capital of Chile. With a mild and rainy climate, the area is covered by native forests of high ecological significance. Volcanoes, lagoons, and natural water streams complete the natural landscape of the region. It is the ancestral territory of the Mapuche people, and many communities in the region struggle against the expansion of productive activities on their land. From the 1990s onward, the area has experienced rapid urban and population growth, accompanied by the expansion of forestry, aquaculture and agriculture. Mass tourism has grown significantly, making the lakeside cities of Pucón and Villarrica the most important tourist centers in Southern Chile. The arrival of artists and lifestyle migrants in the last 20 years and the development of alternative life projects have given rise to a diverse and multifaceted society and culture (Zunino and Spirito, 2023).

The population and economic dynamics in the area have led to a steady increase in the pollution of the waters of Lake Villarrica. Recent studies suggest that this pollution stems from productive activities, such as aquaculture and tourism-related enterprises, which discharge contaminants directly into the lake. This situation is further aggravated by the lack of sanitation infrastructure in many rural areas (Escobar, 2019; Simon and Ceballos, 2023). Pollution becomes perceptible to the population mainly during summer through odor emissions and changes in the coloration of the lake. The deterioration of the environment has jeopardized tourism activities and raised concerns among various local and regional actors, prompting the mobilization of numerous individuals in a collective effort to protect the waters of the lake. Several social collectives of diverse inspirations are part of the struggle, acting independently and converging in events that call for a common response to the problem. They have undertaken various actions, including demonstrations, artistic interventions, the development of alternative proposals, public complaints, and disruption of urban development projects (Goich et al., 2021; Sánchez et al., 2018). To control emissions and the disposal of contaminants, the state apparatus responded with administrative and economic incentives. In early 2025, the process culminated with the approval of a “Decontamination Plan.” The mandatory social participation process required for the elaboration of the plan was conceived in technical and analytical terms, strictly defining the different phases of the process and the information considered in decision-making, generating multiple tensions between local authorities and organizations active in the protection of the waters of the lake (Goich et al., 2021). Thus, the decontamination strategy promoted by the State has not mitigated the environmental degradation process of the lake, and criticism has arisen regarding the rigid and centralized nature of a governance system that operates with technical criteria and grants property rights to water users (Simon and Ceballos, 2023).

In this research, we take the defense of the waters of Lake Villarrica as an epistemic moment to explore, together with the defenders, other ways of knowing based on the principle of human interconnectedness with the world. We are persuaded that the tools derived from the classical mode of thought are insufficient to address modern problems such as environmental degradation in its depth (Escobar, 2015). Thus, instead of focusing on technical-structural aspects to find a “solution to the problem” through evidence and argumentation, this investigation seeks to create a broad and collaborative space for first-person dialogue, fostering deep reflection and the emergence of new impulses for collective action. Our approach involves engaging in collaborative exploration that aligns with research practices aimed at acquiring contextual understandings. Instead of confining the analysis to the pursuit of objectified knowledge, this perspective embraces the ambiguity inherent in social interactions and fosters dialogue among participants (Johansson and Linde, 2005). The collaborative research process stimulates collective thinking and combines different types of knowledge and skills using creative and imaginative synthesis tools to generate novel responses (Brown, 2015; Roderick, 2020). Thus, this research practice inherently involves an exploration aimed at uncovering new insights, with the resulting knowledge emerging from the process of social interaction itself.

In this collaborative exploration, we bring to empirical experience the ideas developed by thinkers such as Deleuze (1994), Goethe (2007), and Merleau-Ponty (1993), who have recognized, from different perspectives, the profound connections between human beings and the world. Their insights allow us to establish a space of inquiry that lies between what is presented to our physical senses in our immediate experience and the forces that sustain the becoming of the world. Deleuze inspires us to view human creativity as a central element for accessing a virtual field of infinite possibilities of which we are part. Goethe guides our investigative practice toward the inner contemplation of nature, revealing the formative forces or ideal principles active within it through the faculty of imagination. In turn, Merleau-Ponty leads us to pay attention to the body as a mediator between human beings and the meanings of the world.

Various research tools can help us experience the connection between the embodied subjects and the world. From a feminist perspective, qualitative techniques such as stories, observations, and narratives have been employed to explore bodily sensitivity and lived experiences (Barbour, 2016; Chadwick, 2017). In Latin America, the body-territory perspective uses sensitive cartography to examine the relationship between human bodies and their natural, social, and political environments (Guzmán, 2025; Zaragocin and Caretta, 2020; Zaragocin, 2024). In research-creation, artistic expression and imagination are utilized to generate knowledge beyond the textual, acknowledging the full epistemic power of human sensitivity (Loveless, 2019; McCormack, 2008; Palma-Irarrázaval, 2022). Body movement has been employed to study how people resonate with the forces of the world through dance and body movements (Rabadán, 2019; Semetsky, 2004). From the ecology of perception, work is conducted with sensory experiences that intertwine with ecosystems, fostering a sense of reciprocity with animals, plants and objects (Abram, 1996). These tools offer a wide range of possibilities for our exploration. Given the researchers' experience in previous collaborative work and their familiarity with these diverse techniques, we privilege the use of artistic expression and body movement as methodological tools to facilitate the connection between bodies and the world. Additionally, reflexive conversation plays a central role in the process, functioning as a social research tool that facilitates the collective observation of events and the creation of dynamic spaces of interaction that are open to the emergence of novel ideas and emotions (Kaplan, 2015).

In July 2022, the researchers distributed a broad invitation to participate in a collaborative workshop to exchange perspectives on the degradation of water and envision ways to project the collective struggle into the future. This invitation was directed to organizations defending the waters and was distributed through social media to broaden the scope of participants. More than 30 people expressed interest in participating, and we exchanged thoughts and impressions with many of them regarding our motivations, how we envisioned the workshop activities, and the necessary commitment in terms of time and dedication. Ultimately, a group of 12 people, in addition to the researchers, was formed. The group includes people with diverse backgrounds and perspectives, including environmental activists, leaders from Indigenous and non-Indigenous communities, individuals affiliated with traditional political parties, and members of cultural and artistic collectives. Gender representation was equal, and the age range fluctuated between 24 and 72 years old.

The exploration brought together people actively involved in negotiations with the state, alongside others mobilized in defense of the waters or interested in contributing to this cause from their own frameworks of understanding and expression. Many participants were already familiar with each other because of their previous participation in collective actions. Although they are not part of a common organization, they identify themselves as part of a broad and diverse movement committed to protecting the waters of Lake Villarrica. Past collaborative experiences with them reveal that this movement is composed of small collectives operating on assembly driven logic, lacking a defined hierarchical structure, and featuring multiple leaders and spokespersons who represent either themselves or others. When significant events occur, such as the approval of regulations or the initiation of extractive activities, the group rapidly mobilizes through social networks to engage in large-scale collective actions. We conducted this study between October and November 2022, during a period of high social and political unrest related to the Chilean social uprising of 2019–2020 and the failure of the constituent assembly that followed (Frei, 2024). At this time, lake pollution was a widespread issue, and state agents were actively formulating a “decontamination plan” and implementing the citizen participation process as required by law.

In this collaborative research, the researchers were active participants who fully engaged in all exercises, discussions, debates, and social interactions. From the beginning and throughout the entire process, we openly acknowledged and shared our positionality with the other participants. The authors maintain a critical perspective on technocratic and rationalist forms of knowledge and, from this standpoint, have facilitated discussion panels and collaborative research with various human communities affected by prevailing development models. As university-affiliated researchers, we operate within an institutional context that upholds conventional research practices that make people objects of research. Many communities are understandably cautious about engaging in academic research. Establishing the trust essential for collaborative efforts is a significant undertaking that necessitates delicate and honest dialogue for mutual recognition, both during the formation of the group and throughout the project's duration.

To design the tasks and determine what to do, how, and when, we began by vividly reviewing the concepts that guide the research (virtual plane, ideal principles, formative force, imagination, body-world synchronization), envisioning how we could apply them with the group. After evaluating our limitations and capabilities, we determined the tools we would employ and the potential sequence for their deployment. In the first workshop, we agreed to emphasize reflective conversations about past events that had led the participants to the present moment. In the second workshop, we chose to focus on the diverse experiences, reflections, and emotions accompanying the ongoing struggle to defend water. In the third workshop, we decided to open the conversation on the future of water defense, using artistic expression through drawing as a tool for stimulating imagination and active thinking. This approach creates a progression from the past to the future, facilitating a step-by-step process of knowledge construction. Given the interest it generated and its effects on the group, body movement became an important tool used in all three sessions. In addition, poetic expression emerged spontaneously as a means of expression during the second workshop. Throughout the three workshops, reflective conversations provided a platform for participants to share their thoughts, interpretations, and sensations as events unfolded (Table 1).

**Table 1:** Research guidelines.

Dimensions	Workshop 1	Workshop 2	Workshop 3
Temporal perspective	Past	Present	Future
Leading question	What events and experiences have brought us to the present point?	How do we experience the struggle to protect water?	How can we project the defense of water into the future?
Tools	Reflective conversation, body movement	Reflective conversation, body movement, artistic expression through poetry	Reflective conversation, body movement, artistic expression through drawing

This collaborative exploration approach is characterized by its flexibility, allowing emerging events to shape the process as they unfold. Within this framework, researchers actively participate, fully engaging in all exercises, discussions, debates, and social interactions. We created a comfortable and welcoming environment to encourage first-person reflection and the expression of diverse sensibilities. The room was equipped with various amenities, and at its center, we arranged a conversation circle with 16 comfortable chairs; this setup was maintained throughout the three workshops. As the activities progressed, social interactions intensified, requiring researchers to remain attentive and intervene only when necessary to maintain the flow of conversation and provide guidance for distinct practices. After each significant event, the researchers and all interested parties engaged in open discussions about the implications of the occurrences. These preliminary findings were shared openly throughout the process and juxtaposed with diverse perspectives in an iterative process that was highly dynamic and receptive to various outcomes and revisions. In the following section, we will detail the events that transpired in the three workshops, which we characterize as “Difference in Motion,” “Intertwining with Water,” and “Hope and Future.” Subsequently, we examine specific moments of inflection that mark the emergence of something new in the collective space.

## 4 Description of events

### 4.1 Workshop 1: difference in motion

The first workshop began with a conversation on the purpose of this collaborative research and the positionality of the researchers. Each of us shared our motivations, interests, and perspectives on world events and academic life, emphasizing how our current work aligns with our individual journeys. Gradually, mutual trust began to take shape. To guide the dialogue toward the defense of water, the researchers encouraged reflection through two questions: Who am I? How did I reach the place I am at now? These broad questions, which do not have immediate answers, are intended to foster an atmosphere that encourages introspective reflection on the significant experiences that have shaped current positions and actions in the defense of the waters. Initially, these questions appeared unexpected, resulting in a series of fragmented and incomplete statements. Gradually, people began to present themselves naturally, acknowledging and highlighting their viewpoints, positions in society, and feelings and emotions about what was happening to water. The conversation then shifted to past experiences in defending the waters and the motivations for participating in this exploratory work. The conversation gradually gained momentum as each participant reacted to what they had heard previously, and a common feeling of defeat, powerlessness, and hopelessness emerged. Emphasis was placed on the failure of the goals pursued during the 2019–2020 uprising, the existing social and cultural inequalities, the burden of bureaucracy, and the feeling of always arriving late to important discussions. The atmosphere was permeated with feelings of defeat.

**Figure 1:**
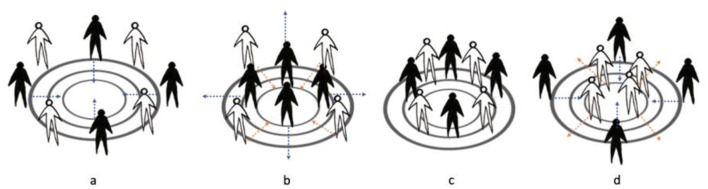
Embodied exercise “Circles in Motion”. Sequence a, b, c, and d. Source: authors.

Following this discussion, the researchers concurred on the necessity to modify the prevailing pessimistic dynamic, which appeared to be leading to a standstill and fragmenting the social body. At this juncture, one of the researchers suggested using a body movement exercise that had been helpful in similar situations. The intention was to stop abstract reflection and guide attention toward the sensations of the body when it moves, evoking reflection to arises from the experience of movement. We invited the participants to form a circle. Once formed, half of the group alternately began moving toward the center and then returned to their original position, maintaining a constant flow (Figure 1a). Those remaining in the initial circle began their own movement upon seeing others return from the center. In this back-and-forth movement, a new circle encompassing all participants is created in a place different from the starting point (Figure 1c). For a fraction of a moment, this circle seems to stop, only to dissolve and reappear in the next reverse movement. The movements continue as the position of each one of us continually inverts (compare Figures 1b, d).

Initially, this exercise generated frustration and discomfort because of the difficulty in maintaining the rhythm and form. However, we gradually managed to synchronize our movements, generating enthusiasm and surprise. Once this synchronicity was reached, a new complexity was introduced to the exercise: clapping when a new circle forms. As before, this required a new process of adjustment and coordination. Surprisingly to everyone, circles appear and disappear in movements that sometimes unite some, sometimes all. The circle never acquires a definitive shape and is in perpetual motion.

As the bodies were in motion, we playfully experienced different speeds and rhythms. At that point, reflection was stimulated by one of the researchers posing with a loud but friendly voice: What do we place at the center of our movements in defense of water? This reflective prompt was intended to encourage active reflection as we collectively experienced the movement, stimulating thoughts from within the movement itself. Three broad themes emerged from these conversations. First, social organization was seen as a catalyst, echoing what happens at the social level. Second, the value of remaining open to change and the relevance of emotions as catalysts for action were highlighted. Third, many valued the bonds of solidarity and respect that can be created between people and the importance of social encounters. These elements gave rise to the reflection that the individual is always part of something, in a constant relationship with others.

The session ended with three questions to nourish the thoughts for our next encounter: What drives us to defend water? What forces lie behind the events or milestones that have brought us to this moment? Can external factors be distinguished from internal factors? These questions point to the deeper aspects of life that usually remain unnoticed in our interactions with the world.

### 4.2 Workshop 2: intertwining with water

We initiated the second workshop by reflecting on the territory that provides us with shelter and hosts all the relationships we cultivate. Numerous references were made to territorial transformations and the injustices inflicted on both nature and culture. Anger and longing are intermixed in various ways. At this point, the group insisted on repeating the exercise “Circles in Motion.” Once the group reached a very precise synchronization and maintained it for a long period, one researcher invited the participants to imagine the straight line and triangles traced when they walked forward and backward. When asked what happens when someone moves forward, ideas associated with leadership, direction, and taking action arose; when moving backward, notions of pausing and openness to allow others to lead emerged. The multiple and invisible relations that emerge while in motion stimulate the recognition of the importance of each individual movement in maintaining the circular form.

The session continued with a reflection on the two questions posed at the end of the first workshop: working first individually, then in groups of three, and finally, in a plenary session. Previous experience from the researchers suggests that it is beneficial to begin by making the individual position conscious and clear, and then allow it to converge with other positions, thus enabling its transformation and gradual convergence with the collective. This process is not without tension and generally culminates in rather intense group discussions. The plenary conversation that began in a friendly and approachable manner took on a more critical tone when one participant introduced the notion of “power.” It emerged as an evocation of a force that permeates the social body and closes all other alternatives and possibilities for action. The idea was concretely expressed when a participant vehemently placed a plastic bottle in the center of the circle, symbolizing how water has been transformed into a mere commodity and making visible the antagonist: the power represented by large corporations and the rich and powerful people. Thus, this symbolic expression is not restricted to the domination of nature but represents the domination of life itself, setting in motion a process that encompasses nature, society and individuals. The symbol at the center is an image of the world trapped inside a bottle, caught within modern capitalism. The conversation turned into a dichotomous debate. On one side, those arguing for the dismantlement of capitalism, and on the other, those emphasizing alternative ways of living and the development of a different consciousness. The positions hardened and tensions grew. The possibility of dialogue seemed to be closing down.

In this tense atmosphere, one of the participants suggested acknowledging the “power of water.” This imaginative and spontaneous intervention marked a turning point in the mood of the group. Water, now conceived as an agent with its own voice and strength, opens a different, gentler, and more inspiring horizon for discussion. During this conversation, one of the participants read the poem “Las aguas, fuentes de vida” (The Waters, Sources of Life) by Argentine poet and artist Arjona Delia. The poetry acted as a mediator, redirecting the dialogue toward what seemed more essential: the sacredness of water and the need to protect it from exploitation. Confrontation gave way to a space of listening as ideas were shared about the need to end the “permanent struggle” that wears down both the individual and the collective. The urgency for immediate results and the importance of creating spaces for real human encounters were also mentioned. Reflection on our own shadows and contradictions led to the reflection “we are what we do with water” and a critique of the “ego of the activist.” These comments about the attitudes of water defenders stem from the frustrations that the group has experienced in recent years. However, this is offset by an attitude that evokes the power that arises from the people themselves. One of the participants summarized this sentiment by saying, “They make us believe we have no power, but collective power does exist.”

In this second workshop, we reflected on the current situation faced by water defenders. To prepare for the transition to the third workshop, which focuses on looking toward the future, we are leaving two reflection questions to work on in the meantime: What do we let go of? What do we hold onto? These open-ended questions were designed to encourage individual reflection, so that each perspective could be more strongly integrated into conversations during the last workshop.

### 4.3 Workshop 3: hope and future

The session began with all participants seated in a circle, revisiting the concept of power and the challenges in the prevailing social and political context. A body-movement exercise followed, inviting participants to walk forward and then backward, connecting their movements to the sensations that emerged. Walking forward evokes action, focus, and decisiveness, whereas walking backward brings perspective, doubt, and openness to uncertainty. Next, we attempted to form a rhombus with our bodies, an exercise that proved challenging because of the difficulty in achieving that shape. In contrast, when we tried to form a circle, the group accomplished this quickly and easily, prompting reflections on the “common image” underlying each shape. One participant spontaneously asked what defines a triangle as a triangle, creating an opportunity for participants to visualize that there is always an element that unifies all specific manifestations of a triangle. This imaginative dynamic facilitated reflection on the importance of diversity, recognizing that, like a triangle, a group can bring together different voices as long as a common purpose exists. This led to a transformation in the tone and meaning of the conversations, which became less confrontational and more open to the opinions of others.

We then worked with our sensitivity and imagination through drawing and poetic narratives. The invitation was to reflect on the three states of water and how, in its ceaseless transformation, there is something that always remains. First, we worked individually, then in small groups of two or three participants, and culminating in a plenary session. During individual work, we contemplate the relationship between our bodies and the watery element, bringing our memories, pains, inspirations, and reflections to consciousness. We then imagined the movement of water and expressed our thoughts and feelings in narrative form. Subsequently, in small groups, we shared our creations and narrated their significance to each other. We concluded with a collective conversation in a circle, sharing insights about our experiences. During this dialogue, we engaged in profound reflection on the multifaceted nature of water, recognizing it as more than a mere resource. We acknowledge its diverse forms of expression and its perpetual movement across the globe. We share insights about the properties of water and the various lessons it imparts to humans. How far are we from living in relation to the power of water?

The drawing exercise set the stage for the afternoon session, which focused on projecting into the future by revisiting the questions: what do we let go of? What do we hold onto? Various interventions pointed to the need to release inconsistencies, feelings of failure, and the tendency to blame others. We identified a set of emotions and habits that undermine collective action and lead to the illusion of being “saviors of the world.” These criticisms of the water defenders themselves embody the frustrations of a seemingly fruitless struggle over the years. However, they also contain an openness to new approaches to the future, no longer anchored solely in a critique of capitalism but also in the strength that the group acquires when it recognizes its interconnectedness with water and the power it holds. We can symbolize this attitude with the words of one of the participants at the end of the workshops: “We are all the waters of the basin.” The session concluded in a silence charged with longing and hope. Although we do not yet know how to solve this problem, a quiet joy has emerged, along with the desire to continue working together.

## 5 Discussion: becoming, sensitivity, and imagination in research

In this research, we bring to empirical experience a form of knowledge grounded in the contributions of Deleuze, Goethe, and Merleau-Ponty. We understand the act of knowing as a concrete event that arises when human beings encounter the world and experience the forces it contains. Our approach directs the focus to how participants experience a virtual realm of infinite possibilities (Deleuze), activate their imagination (Einbildungskraft) to visualize the ideal forces at work through them (Goethe), and synchronize their bodies with the world (Merleau-Ponty). These theoretical constructs will guide us in examining the events that transpired, focusing on moments of inflection where something new emerged in the collective realm.

The collaborative work opened in an atmosphere of confrontation, where each position struggled to prevail over the other. The tone was vehement and categorical. Multiple paradoxes and ambiguities permeated the narratives, and divergence resonated strongly in the mood of the group. While some positions, with different emphasis, called for the end of capitalism, others advocated the development of a more fraternal and ecological consciousness and practice. A centralizing and hierarchical style of thinking dominated, based on a transcendental model of reality in which diverse theories, concepts, and causal relationships are drawn from an abstract sphere, detached from lived experience, and presented as “explanations” of the phenomenon. Deleuze and Guattari (2004) characterize this way of thinking as arborescent, suggesting that it is the conventional way of constructing and understanding knowledge production.

The exercise “circles in motion” proved particularly effective in interrupting these thought patterns. At the beginning of the movement, participants felt the pressured to be exposed to the gaze of others as they tried to maintain rhythm and precision. Gradually, the sensation of horizontality and belonging intensified as the bodies united in a continuous flow of intensities, driven by a force that no longer emanated from individual bodies but rather from the periphery of the world. Thus, this exercise appears fundamental for the emergence of embodied ways of knowledge, revealing how movement unfolds through reciprocal attunement between the body and the world, in a similar way that dance does (Merleau-Ponty, 1993). This parallels also the example of the surfer riding the wave used by Deleuze (1995) to depict the virtual plane of intensities and pure movement. During the exercise, we stop thinking about the movement and begin to think within the movement, activating a more flexible and receptive way of thinking that is open to new insights.

**Figure 2:**
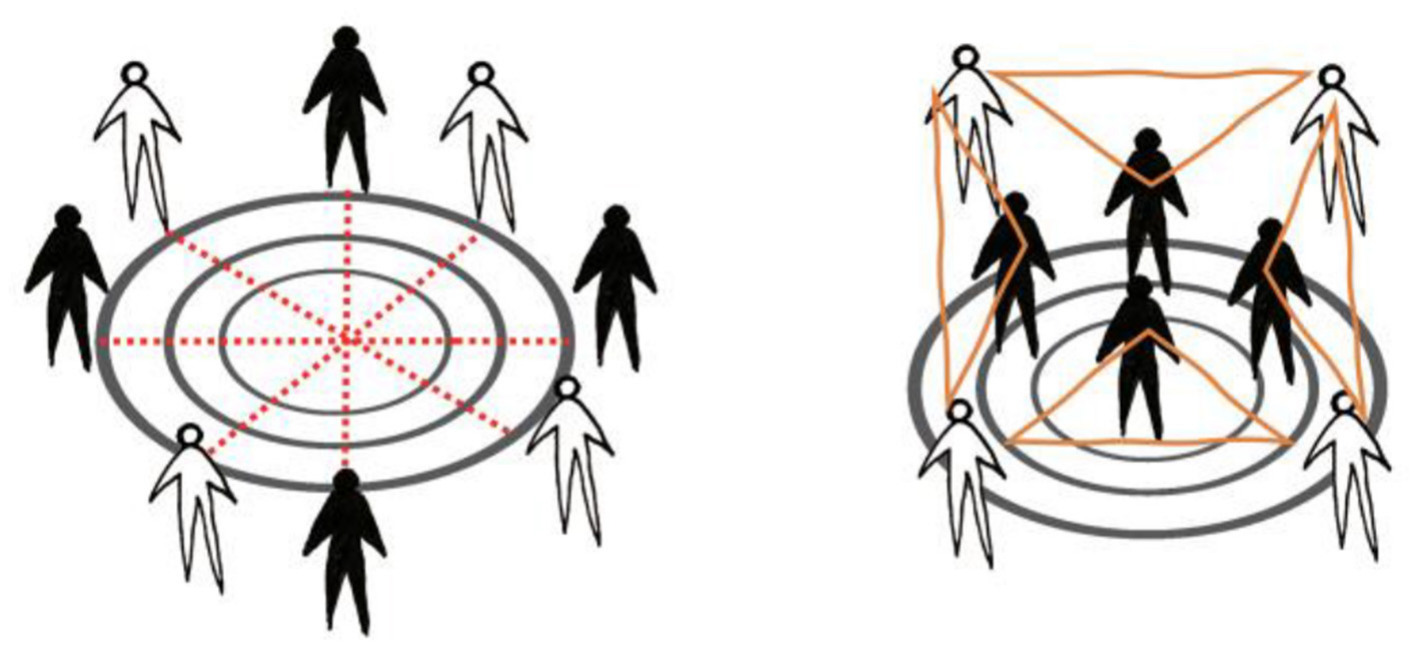
Triangles appearing and disappearing in the exercise “moving circles.” Source: Authors.

This exercise was repeated several times. At one point, one of the researchers intervened, directing attention to the triangles that appeared and disappeared among the participants. As they moved, the participants quickly recognized these shapes and the different connections they established between them (Figure 2).

When the participants became aware of the different triangles appearing and disappearing, they sensed an ideal form emerging through their own bodily movements. This ideal form is not an object we can find behind or beyond the phenomenon in a transcendental sphere; rather, it only comes into presence within the individual parts and is identical to the multiplicity of the phenomenon (Böhme, 1986; Bortoft, 1996). During the movement, this universal acquires a perceptible reality only through the movement of bodies in accordance with the instructions provided by the researchers. The moment of perception of this ideal form within the movement itself was a surprising event, leading to the realization that different individuals can enter a collective movement while retaining their uniqueness. The participants became part of an all-encompassing collective movement, which transformed their attitude toward the work. They realized that those at the forefront are never truly alone; behind them are those who provide support and are part of the same collective effort. As the movement progresses, this support appears in its opposite form, revealing the relationship of reciprocity among them. This fleeting yet intense experience of the forces active in the world manifesting in the movements of the bodies was accompanied by feelings of joy and delight, creating a more favorable environment for collaborative work as participants realized their mutual dependency and the capacity of diverse people to work together.

This exercise was followed by a conversation that led to the further realization that, alongside the ruthless power of capital, another form of power originates in the collective realm and is connected to the wholeness of the world. As one participant observed, when reflecting on the structural power of capital, there exists another form of power that encompasses us all and remains concealed from our awareness. In a similar vein, Hathaway and Boff (2014, p. 121–123) argue that imagining other possible worlds requires reconsidering the notion of power. This involves shifting the focus from the overarching structures of domination to a “power-from-within,” which is understood as a form of power emerging from the inherent strength of collective processes. In addition to this form of empowerment, they also identify a “power with,” which denotes a form of power arising from the interdependence of human beings with the wholeness of the world (see also Garcés, 2013; Kaplan, 2005, 2015).

In the second workshop, poetic language emerged unexpectedly, disrupting the course of events at a time when divergent opinions were fragmenting the social body. The rhythmic element of poetry opens the feeling life, inviting silence and reverence, soothing passions, and creating a space for the content of the world to manifest within the group. It provided a pause in the relentless ebb and flow of opinions, altering the collective disposition and enabling the coalescence of different perspectives in a common stance: the recognition that the watery element, in its many forms and constant transformation, reveals a power intrinsic to the world and imparts ethical principles, rendering its presence worthy of defense. In this manner, poetic imagination facilitates an experience of knowledge intertwined with our emotions, acting as a mediator between the human and the nonhuman (Pulvirenti and Gambino, 2013).

**Figure 3:**
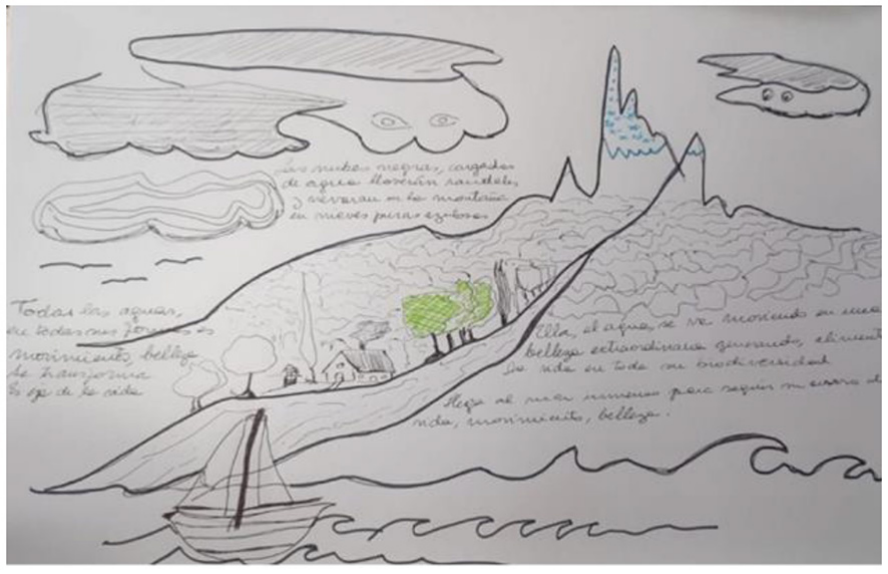
States and movements of water. Visual testimony of the work developed by one participant. Courtesy of the artist.

The irruption of poetry had a natural continuation in the drawing exercise practiced in the third workshop. Water emerged as a living concept that leads us to rethink our relationship with the world. Thus, in this more-than-human world, water becomes a collaborator in what we know, imparting important lessons to us. Indeed, for Neimanis (2017a,b), the sensitivity and intelligence of water reveal patterns of existence that follow certain “hydro logics.” Water gestates, dissolves, communicates, conducts, remembers, sculpts, and differentiates, challenging every attempt by classical thought to contain and define it from a single perspective. Thus, the imagination of water arises at the meeting point between the inner and outer worlds (Gosetti-Ferencei, 2025). Water is imagined as weaving across planes, revealing its vital and transformative capacity, and uniting the natural, social, human, and non-human realms (Figure 3).

**Figure 4:**
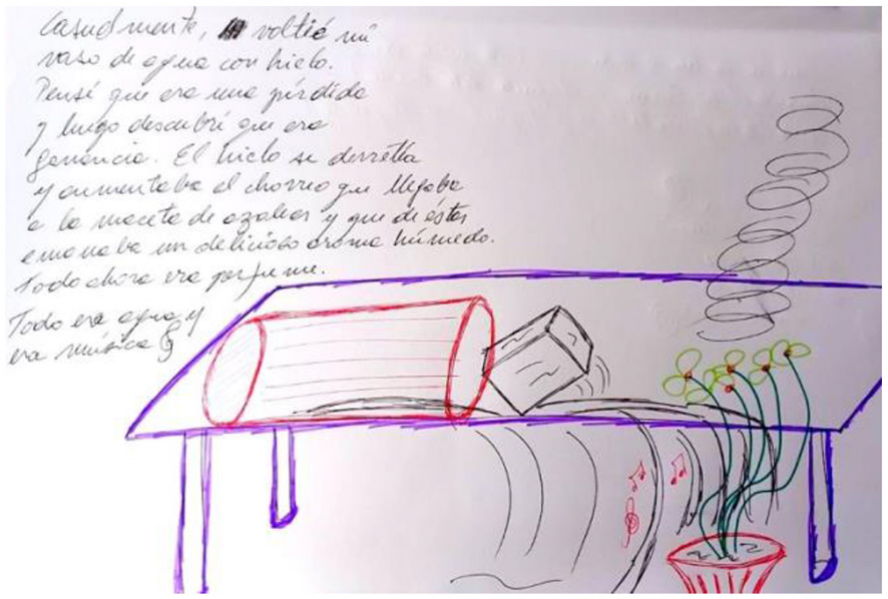
Azalea flowers nourished by water. Visual testimony of the work created by one participant. Courtesy of the artist.

Further, one participant used the following narrative in the drawing:

*I thought it was a loss, but then I realized it was a gain. The ice melted and increased the flow of water reaching the azalea (flowers) pot, which gave off a delicious, moist aroma. Everything was now perfumed. Everything was water and music*. (Figure 4, translation authors)

An apparent loss is transformed into a generative flow that nourishes the azaleas, suggesting how water silently reconfigures and transforms the relationships between humans and the world. Thus, water reveals itself not as a passive substance but as a creative force and active collaborator, turning accidents into events through which we can experience our rootedness in the world.

These imaginations were powerful in reshaping the groups relationship to the becoming of the world. According to Hüppauf and Wulf (2009), imagination allows us to complete what we perceive with our bodies, giving our perceptions coherence and meaning. This meaning is not something transcendent; rather, it is intertwined with our sensory experiences, memories, and emotions. Therefore, imagination challenges traditional dichotomies such as mind-matter, mind-body, and object-subject, navigating the space between the visible and the invisible and between what is currently present and what is yet to emerge. In Deleuzian language (Deleuze, 1994), imagination is situated on the threshold between the virtual and actual planes, where everything existing in a state of potential meets what is realized in action. Working with imagination facilitates a mental and emotional state that enables us to realize our rootedness in the world more vividly (Merleau-Ponty, 1993). Thus, imagination, guided by the sensitivity of the body, serves as a crucial instrument for experiencing becoming and entering a creative and collective space.

The exercise of “circles in movement,” the emergence of poetry, and the practice of artistic creation activated the faculty of imagination and allowed participants to experience how bodies intertwine with the becoming of the world. Although these instances were unstable and fleeting, they facilitated the emergence of creative moments in which participants could observe the world from a different perspective and open themselves to the collective realm. Throughout the workshops, the group underwent a movement from a conglomeration of disconnected parts to a collective formation in which the parts dialogue, recognize one another, and are able to identify common impulses toward the future. An important support for this new disposition is provided by the experience of the watery element and its becoming. Water ceases to be an abstract and external object toward which we direct our efforts of defense and protection; it becomes a vital and transformative force that embodies its own intelligence and logic within us, making it an essential part of the struggle to safeguard it from depletion. This collective realization allows us to project the defense not only based on a critique of capitalism but also from convictions anchored in our experiences with the watery element in its profundity. This conviction makes the defense of water a valuable and necessary task. At this juncture, diverse singularities converge in flow full of creative potential, enabling different struggles to coalesce into a broader and more intense movement.

The workshop ended at this point, with the feeling of having opened a small window into the future. The group now has different possibilities for projecting itself into the future, depending on the aims and purposes that people themselves establish in their relationship with the world. Thus, the social construction achieved is not a fixed outcome but rather a process that must be sustained through social interaction itself. We did not reach a solution, a consensus, or a new social formation to advocate for water. However, we may have gained something even more valuable: the group rediscovered the motivation and strength to persist in the efforts to defend water.

## 6 Final remarks

To explore collective ways of understanding based on the becoming of the world, we engaged in collaborative work with people who dedicate their time and energy to defending water from the destructive processes it faces. In the three workshops, we used the sensory capacity of the body and the faculty of imagination to experience the world resonating within us. Grasping the deeper meaning of water leads to adopting a way of thinking that no longer interconnects abstract concepts but flows according to images we form that are connected to the movement and rhythm of the world. This approach allows us to glimpse the realm in-between the objective and subjective, where creative thinking unfolds its activity. From different angles, this realm of thinking and feeling has been portrayed by Deleuze, Goethe, and Merleau-Ponty.

As we experience the relationship our bodies establish with the world, we immerse with our thoughts and feelings into a collective sphere imbued with creative potential and multiple meanings. During the work, we partially entered into this connection and openness at specific moments that were charged with intensity. These singular moments were crucial and transformative for the group, enabling vibrant engagement with the inherent logic and forces of water, recognizing its intrinsic power, and making its defense and protection a necessary and valuable collective endeavor. This realization provided the group with a common evolving center, allowing different meanings and struggles to coalesce and thereby strengthening the sense of the collective. In this way, we were able to move from a social conglomerate composed of fragments to a collective formation in which participants recognize one another and are able to identify common impulses toward the future, finding some hope in a time marked by pessimism and defeat.

However, the collective arrangement that gradually takes shape during the work is a construction sustained only through the living interaction among diverse individuals who, in a singular moment, recognize an impulse to work together. Thus, these achievements remain inherently partial and incomplete, highlighting the fragility of these moments when the strength of the collective appears. Indeed, collective constructions based on innovative modes of thinking are vulnerable to rigid and individualistic mindsets, institutions that impose fixed analytical categories, and myriad disciplinary powers that permeate the social fabric. This poses the challenge of persistently renewing the collective constructions reached through the actions of individuals, ensuring that collective impulses continuously adapt to ever-changing contextual conditions.

This study demonstrates that novel ways of thinking, feeling, and acting are not only possible but also within the reach of individuals who choose to work together in this direction. Along with people defending the waters in southern Chile, we opened a small breach in the sea of contemporary abstractions, suggesting an approach to connect livingly to the social and natural worlds. This endeavor requires interrupting habitual modes of thinking and opening ourselves to experiencing the forces intrinsic to the world that permeate human bodies from all sides.

## Data Availability

The raw data supporting the conclusions of this article will be made available by the authors, without undue reservation.
